# The impact of vitamin D on atopic disorders: assessing evidence for a causal relationship

**DOI:** 10.3389/fnut.2025.1584818

**Published:** 2025-04-10

**Authors:** Valeria Andrea Zúñiga, Blanca Bazan-Perkins

**Affiliations:** ^1^Instituto Tecnológico y de Estudios Superiores de Monterrey, Escuela de Medicina y Ciencias de la Salud, Mexico City, Mexico; ^2^Instituto Nacional de Enfermedades Respiratorias Ismael Cosío Villegas, Mexico City, Mexico

**Keywords:** atopy, vitamin D, T helper 2 cell, dendritic cells, regulatory T cells

## Abstract

Since the beginning of COVID-19 pandemic, there has been a noticeable increase in the consumption of vitamin D. Evidence accentuates the generation of a pro-tolerogenic T helper 2 cell state with vitamin D, suppressing T helper 1 inflammatory response. T helper 2 cell polarization is characteristic of atopy. However, although the literature on vitamin D and atopy has yielded controversial results, multiple studies have described an inverse relationship between vitamin D levels and the severity of atopy, as well as an improvement of the pathology with vitamin D supplementation. A different approach is offered in the analysis of the immunological mechanisms by which vitamin D acts in the human body, supporting its use as a promoter of homeostasis. In this sense, vitamin D promotes a balanced state through the action of regulatory T cells, controlling cytokines, both pro- and anti-inflammatory, and by reducing B cell prolif eration and differentiation, thus preventing the possible development of atopy.

## 1 Introduction

Coronavirus disease (COVID-19), caused by the SARS-CoV-2 virus, is an infectious disease that has posed significant threats to global public health and resulted in millions of deaths worldwide (1). Research indicates that a cytokine storm in COVID-19 is linked to higher mortality rates and clinical worsening, with severe cases being attributed to elevated levels of interleukin (IL)-1, tumor necrosis factor (TNF)-α, interferon (IFN)-γ, and IL-6 (2).

Vitamin D deficiency is a widespread global health issue, with an estimated 50% of the world’s population experiencing insufficient levels (3). This has led to a rise in the use of vitamin D supplements. While the risk of toxicity from excessive intake is rare and often underestimated, severe complications such as hypercalcemia, delayed teething, late walking, bone demineralization, and pain have been reported as potential effects of vitamin D toxicity (4–6). Since the beginning of the COVID-19 pandemic, studies have suggested a link between vitamin D deficiency and a higher incidence of infection (7, 8) as well as increased severity of COVID-19. As a result, the sale of vitamin supplements, including vitamin D, has surged during the pandemic due to its potential prophylactic or therapeutic benefits (9).

## 2 Vitamin D’s biological role

**FIGURE 1:**
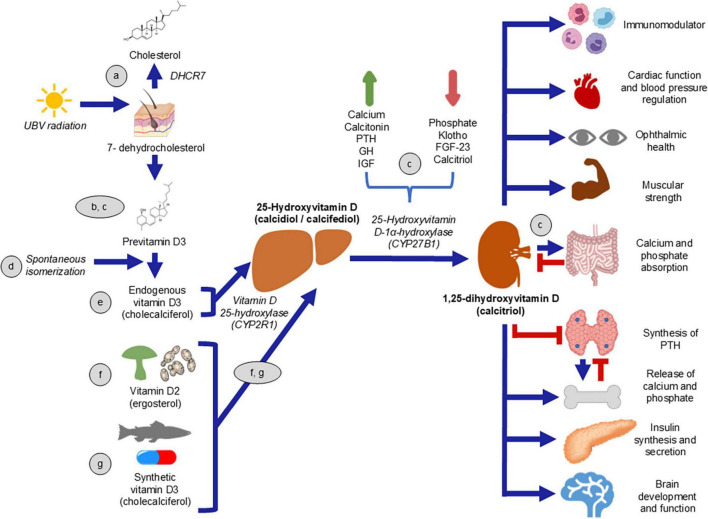
Vitamin D (VD) synthesis and its effects. VD is obtained through the skin production or by alimentary consumption. a) 7-dehydrocholesterol, which can be transformed to cholesterol (24); b,c) converts to previtamin D3 (25, 26) through ultraviolet B radiation. d) Via spontaneous isomeration (24), precholecalciferol changes to cholecalciferol. e) VD can be obtained from food, both of animal (cholecalciferol) and plant origin (ergocalciferol) (27). f) Cholecalciferol is metabolized in the liver to 25-hydroxyvitamin D (28). g) The active form of VD, 1,25-dihydroxyvitamin D, is obtained in the kidneys by the enzyme 25-Hydroxyvitamin D-1α-hydroxylase (which, in turn, is regulated by stimuli). c) Calcitriol has diverse effects on different tissues (29).

Prohormone and vitamin D can be acquired through endogenous production triggered by ultraviolet B radiation, dietary sources, or supplements. Vitamin D plays a diverse regulatory role in the human body (Figure 1), supported by evidence of its receptor being present in various tissues (10). It serves as a key regulator of mineral homeostasis, influencing the parathyroid glands, bones, and intestines. Additionally, vitamin D has a wide range of other biological functions, including significant effects on the immune system (11).

## 3 Vitamin D and immune system modulation

For this reason, vitamin D can influence both innate and adaptive immunity, promoting the induction of monocyte and macrophage signaling, particularly through antimicrobial peptides like cathelicidins and β-defensin 2. Additionally, vitamin D inhibits the activity of B and T cells, reduces levels of pro-inflammatory cytokines such as IL-1, IL-6, and TNF-α, and suppresses antigen-presenting cells like dendritic cells (11, 12). Research indicates that vitamin D shifts the immune response from an inflammatory T-helper (Th) 1-cell response to a pro-tolerogenic Th2 response, halting cytotoxic T lymphocyte infiltration and increasing CD4+ CD25+ regulatory T cells (Tregs) (10). Th2 cells function by releasing mediators and influencing the activity of other cells in the immune system. Th2 cells are a subset of T-helper cells that play a vital role in immune responses, particularly in allergic reactions and the defense against parasitic infections. One of their key functions is the production of IL-5, IL-13 and IL-4, cytokines that are essential for the differentiation and activation of various immune cells. IL-4 not only promotes the differentiation of naive T cells into Th2 cells but also stimulates B cells to produce immunoglobulin E (IgE), which is crucial in allergic responses. Additionally, IL-4 helps in the activation of other immune cells, such as macrophages and eosinophils, further amplifying the immune response. The production of IL-4 by Th2 cells is tightly regulated and is a hallmark of Th2-mediated immunity, making it a critical cytokine in both normal immune function and in the development of allergic diseases (13). This has raised concerns about the potential development of atopy in individuals who consume excessive amounts of vitamin D.

Atopy refers to a predisposition toward an exaggerated immune response to allergens and antigens, characterized by overproduction of IgE (14), state that can be obtained by two types of hypersensitivity, type I and type IVb, of the many described. Type I hypersensitivity, allergens trigger an immune response through IgE and Th2 cells. The sensitization phase involves the activation of antigen-presenting cells, leading to the production of allergen-specific IgE by B cells. Type IVb hypersensitivity, driven by Th2 cells and eosinophils, contributes to chronic inflammation. In this response, IL-4, IL-5, and IL-13 mediate IgE synthesis, eosinophil recruitment, and tissue remodeling. Group 2 innate lymphoid cells (ILC2) amplify the response and contribute to chronicity by promoting eosinophil and basophil recruitment. The interplay between Type I and Type IVb hypersensitivity, the named T2 high phenotype, involves overlapping mechanisms such as eosinophil activation, barrier dysfunction and IgE production, which drive persistent tissue damage and chronic allergic inflammation (15).

Scrutinizing with the aforementioned, vitamin D suppresses the maturation, differentiation, and survival of dendritic cells. It also reduces the expression of major histocompatibility complex type II and costimulatory receptors CD40, CD80, and CD86 (10), which are typically elevated in atopic conditions (16), thereby impairing T cell interaction and activation. Furthermore, vitamin D decreases the proliferation and differentiation of B cells, including their tendency to produce IgE. Additionally, this vitamin promotes the development of regulatory T-lymphocytes (11), which can enhance immune tolerance by increasing the production of IL-10 and transforming growth factor β (16).

### 3.1 Vitamin D and atopic diseases

Debates have emerged about the potential effects of vitamin D and its relationship to the severity of atopic diseases. However, some researches support the notion that insufficient vitamin D levels are associated with heightened atopic dermatitis (AD) severity, and correcting this deficiency through supplementation may lessen symptom severity (17–20).

Vitamin D supplementation demonstrates clinically meaningful benefits for AD, particularly through its immunomodulatory effects on T2 inflammation. Borzutzky et al.’s (18) pivotal randomized controlled trial revealed that high-dose weekly vitamin D (50,000 IU) not only reduced AD severity scores but also significantly lowered IL-13 levels and showed trends toward decreased IgE. These findings provide direct evidence of vitamin D’s ability to downregulate key T2 immunity biomarkers, offering a mechanistic explanation for its therapeutic effects. The immunomodulatory potential is further supported by Ng and Yew’s (19) meta-analysis, which found the strongest treatment effects in patients with baseline vitamin D deficiency, a population known to exhibit exaggerated T2 immune responses. The studies collectively suggest that vitamin D supplementation may help restore immune balance in AD by suppressing Th2-mediated inflammation, particularly in pediatric and deficient populations where this pathway is most active.

The clinical benefits of vitamin D extend beyond biomarker modulation to measurable improvements in disease severity and patient outcomes. Both Ng and Yew’s meta-analysis (19) and Borzutzky’s (18) trial reported statistically significant reductions in standardized AD severity scores with supplementation, with effect sizes comparable to some conventional therapies. Notably, the benefits appear dose-dependent, with Ng’s study showing superior outcomes at ≥4,000 IU/day and Borzutzky’s regimen demonstrating rapid efficacy at pharmacologic weekly doses (18, 19). While Nielsen et al. (20) caution against universal application due to heterogeneous responses, their subgroup analyses still support vitamin D’s utility in deficient patients. From a clinical perspective, these immunomodulatory and therapeutic effects position vitamin D as a safe, low-cost adjunct therapy that may reduce reliance on immunosuppressants in select AD populations, while addressing the common comorbidity of vitamin D deficiency prevalent in AD patients.

**FIGURE 2:**
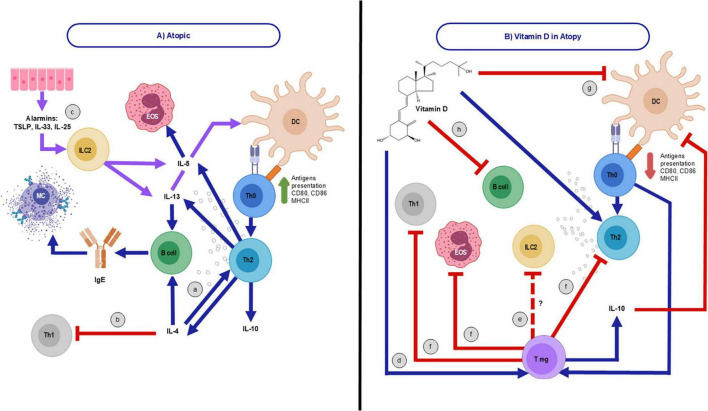
Immunological behavior of atopy as a disease and with vitamin D consumption. **(A)** Immune system in atopy. T helper (Th)-2 cells, an atopy feature, a) release cytokines such as interleukin (IL)-4, that inhibits Th1 cells generation (b), IL-5, IL-10, and IL-13. Likewise, there is a notable elevation of antigens presentation, CD80/CD86 expression, major histocompatibility complex II (MHCII) action, immunoglobulin E (IgE) production, mast cell (MC) release, and eosinophils (EOS) activation. c) Also, group 2 innate lymphoid cells (ILC2) are triggered by cytokines released from epithelial cells, known as alarmins, including IL-25, IL-33, and thymic stromal lymphopoietin (TSLP). Once activated, they secrete significant amounts of type 2 (T2) cytokines, such as IL-5 and IL-13, which further enhance the T2-cell response (15). **(B)** Possible Vitamin D’s (VD) regulatory mechanisms in atopy. d) VD may regulate atopy’s hyperresponsiveness through T regulatory cells (Tregs) differentiation (10), e) possibly inhibiting ILC2 (21) and f) suppressing EOS and impeding Th1 and Th2 functions, which regulates cytokines of the latter. g) Additionally, VD suppresses dendritic cells (DC), h) and B cell differentiation (30), reducing antibody production, including IgE, and antigen presentation.

The prevention or absence of development of allergic or atopic diseases in individuals who consume vitamin D may be also attributed to the role of Tregs. There are various reasons why vitamin D can explain its benefits in atopic diseases, which are heterogeneous in nature. Nevertheless, it is important to mention that the mechanism that can explain the benefit of vitamin D supplementation is related to this vitamin’s immunomodulatory effects that may indirectly suppress IL-4 production by inhibiting Th2 cell proliferation and promoting Tregs function, by which they can possibly decrease levels of ILC2 (21). As previously mentioned, ILC2 cells are known to drive T2 immune responses, which are central to the pathogenesis of atopic diseases. The supplementation of vitamin D appeared to modulate the immune system by diminishing the number and activity of ILC2 cells, thus reducing inflammation and improving the clinical symptoms of AD (18). The latter mentioned, Tregs, can inhibit the proliferation and activation of effector Th cells, such as Th2 and Th17, while also restricting the functions and migration of Th1, Th2, Th9, and Th17 cells, including the production of IFN-γ (16, 18). As a result, Th2 cytokines are suppressed (22) through the possible action of Tregs during atopic conditions, affecting mast cells, IgE, basophils, and eosinophils (22) (Figure 2). Tregs exert their suppressive effects through inhibitory cytokines and signaling molecules like CTLA-4. This receptor plays an immunoregulatory role by preventing the interaction between the CD28 costimulatory molecule on T cells and its ligands, CD80 and CD86. However, recent studies indicate that vitamin D supplementation during pregnancy or infancy does not significantly impact the primary prevention of allergic diseases (23).

## 4 Discussion

Vitamin D plays a critical role in supporting immune homeostasis, acting as a modulator of both the innate and adaptive immune systems. By enhancing the possible activity of Tregs, vitamin D helps to suppress the overproduction of pro-inflammatory cytokines and shift immune responses toward a more balanced state. This not only aids in reducing inflammation but also promotes the production of antimicrobial peptides, providing an enhanced defense against infections.

However, while vitamin D offers several immune benefits, concerns have been raised regarding its excessive intake, particularly in relation to the development of allergic or atopic conditions. Despite vitamin D’s role in shifting immune responses from Th1 to Th2 pathways, which theoretically could contribute to atopy, no concrete evidence has supported this hypothesis. On the contrary, vitamin D seems to promote immune tolerance and reduce the likelihood of allergic reactions by regulating and enhancing Treg activity and cytokine production.

Vitamin D remains a crucial factor in immune regulation, offering potential therapeutic and prophylactic benefits, particularly in the context of infectious diseases. However, its effects on allergic disease prevention and atopy development are still inconclusive, caution should be taken regarding excessive supplementation. As research continues to evolve, public health strategies should focus on maintaining adequate vitamin D levels, ensuring a balance between deficiency prevention and avoiding the risks associated with hypervitaminosis D. Ultimately, vitamin D supplementation should be tailored to individual needs, considering factors such as baseline levels, health status, and environmental exposure to sunlight.

In conclusion, vitamin D supports immune homeostasis by enhancing the activity of Tregs. As a result, the potential development of atopic diseases, which might arise from an assumed rise in Th2 lymphocytes, is likely prevented through the consumption of this vitamin. It is important to mention that the direct mechanism by which ILC2 activity may be decreased by consuming vitamin D is unknown, therefore this panorama should be studied in the future for a better understanding of the possible benefit of vitamin supplementation and atopy.

## Data Availability

The original contributions presented in this study are included in this article/supplementary material, further inquiries can be directed to the corresponding author.
